# ATPIF1 Deficiency Significantly Alleviates *Citrobacter rodentium*-Induced Colitis in Mice

**DOI:** 10.4014/jmb.2604.04015

**Published:** 2026-05-29

**Authors:** Haoyu Yang, Ziqi Li, Dong Yan, Min Li, Lingyun Xu, Yuxin Wang, Genshen Zhong, Minna Wu

**Affiliations:** 1 College of Biological and Chemical Engineering, Changsha University, Changsha 410022, Hunan, P. R. China; 2 School of Basic Medical Sciences, Henan Medical University, Xinxiang 453003, Henan, P. R. China

**Keywords:** Mitochondrial ATPase inhibitory factor 1 (ATPIF1), Infection-associated colitis, *Citrobacter rodentium* (CR), NOD-like receptor protein 3 (NLRP3), Microbiota

## Abstract

Mitochondrial ATP synthase inhibitory factor 1 (ATPIF1) regulates cellular energy metabolism and has been implicated in inflammatory disorders. However, its role in infection-associated colitis remains unclear. This study aimed to investigate the effects of ATPIF1 on host susceptibility and inflammation in a *Citrobacter rodentium*-induced infectious colitis model. ATPIF1 knock out (KO) and wild type (WT) mice were orally gavaged with *C. rodentium* to induce infectious colitis. Body weight, disease activity index (DAI), and colon length, histopathology, barrier function, inflammatory markers, and gut microbiota were assessed using standard approaches, including immunohistochemistry, RT-qPCR, Western blotting, and 16S rRNA sequencing. ATPIF1 deficiency alleviated *C. rodentium*-induced colitis, as evidenced by reduced weight loss, lower DAI scores, and attenuated colon shortening. KO mice preserved epithelial architecture, exhibited increased numbers of goblet cells and ZO-1 mRNA expression, indicating an intact mucosal barrier. Furthermore, KO mice showed reduced infiltration of inflammatory cells, decreased expression of IL-1β and TNF-α, and reduced activation of the NLRP3 inflammasome pathway, although whether this is a direct effect remains unclear. Microbiota analysis also revealed that ATPIF1 deficiency stabilized microbiota composition and reduced pathogenic expansion. ATPIF1 deficiency exhibited a protective phenotype in *C. rodentium*-induced colitis, accompanied by reduced inflammation, preserved barrier function, enhanced pathogen clearance, and stabilized microbiota composition. ATPIF1 may represent a potential therapeutic target in infection-associated colitis.

## Introduction

Ulcerative colitis (UC) is a major subtype of inflammatory bowel disease (IBD), characterized by chronic inflammation of the colonic mucosa, neutrophil infiltration, and ulcer formation. These pathological features result from dysregulated immune responses, genetic predisposition, and alterations in gut microbiota composition [1-4]. Despite significant advances in molecular biology that have elucidated multiple pathways—including cytokines such as interleukin-6 (IL-6) and tumor necrosis factor-alpha (TNF-α), immune cell subsets such as T helper 17 (Th17) cells and regulatory T cells (Tregs), and complex host–microbiota interactions [5-7]—the precise mechanisms underlying UC pathogenesis remain incompletely defined [8].

The murine enteric pathogen *Citrobacter rodentium* (CR) serves as a well-established model that recapitulates key aspects of human enteropathogenic (EPEC) and enterohemorrhagic (EHEC) Escherichia coli infections. As an attaching and effacing (A/E) pathogen, *C. rodentium* infection provokes acute host immune responses, including the activation of inflammatory cytokines and recruitment of leukocytes, which facilitate pathogen clearance [9]. Although *C. rodentium*-induced colitis does not fully recapitulate human UC, it shares key pathological features including mucosal barrier disruption, inflammatory cytokine production, and gut microbiota dysbiosis, making it a relevant and tractable model for studying infection-driven intestinal inflammation [10]. Unlike chemically induced colitis models such as dextran sulfate sodium (DSS), *C. rodentium* infection markedly alters gut microbiota composition and function, indirectly compromising mucosal barrier integrity. Indeed, chronic inflammation in IBD patients is hypothesized to result from aberrant immune responses to gut microbiota, particularly in genetically susceptible hosts. Consequently, infectious colitis models offer critical insights into host pathological responses to enteric bacteria. Emerging evidence further implicates host–microbiota interactions in the regulation of inflammasome activation [11]. Among the immune mechanisms implicated in UC, the NLRP3 inflammasome plays a central role by sensing microbial and danger-associated signals, activating caspase-1, and promoting the secretion of pro-inflammatory cytokines IL-1β and IL-18, thereby amplifying mucosal inflammation [12, 13]. *C. rodentium* infection activates Toll-like receptor signaling pathways, upregulates NLRP3 and pro-IL-1β expression, and enhances NLRP3 inflammasome activation [14]. Thus, the *C. rodentium*-induced colitis model constitutes a valuable tool for dissecting the complex interplay among gut dysbiosis, mucosal immune responses, and epithelial barrier integrity in infection-associated intestinal inflammation, providing important insights relevant to UC pathogenesis.

Mitochondrial ATP synthase inhibitory factor 1 (ATPIF1) is a small (~10 kDa) nuclear-encoded protein localized within the mitochondrial matrix. It modulates the activity of the F1Fo-ATP synthase complex, thereby influencing cellular energy metabolism, with established roles in cancer, cardiovascular diseases, and other pathological conditions [15, 16]. Notably, ATPIF1 exhibits context-dependent functions: it is overexpressed in various malignancies, where it promotes glycolysis and inhibits apoptosis, facilitating tumor proliferation [17]; conversely, during ischemic events, ATPIF1 mitigates cellular ATP consumption, reducing reperfusion-induced cell death [16]. This dual functionality underscores ATPIF1 as a critical regulator of cellular energy homeostasis [18]. In the context of inflammation, mitochondrial dysfunction-induced oxidative stress correlates positively with NLRP3 inflammasome activation, suggesting that ATPIF1, by modulating ATP synthesis, may influence inflammasome-mediated inflammatory responses [19]. Given that mitochondrial function modulates host–microbiota interactions and that gut dysbiosis is closely linked to infection-associated intestinal inflammation, it is imperative to investigate the effects of ATPIF1 deficiency on microbial ecosystems under inflammatory conditions. Previous studies demonstrated that ATPIF1 inactivation ameliorated DSS-induced colitis, although this was limited to a chemical colitis model [20]. Additionally, inhibition of ATPIF1 activity was reported to enhance murine neutrophil antibacterial function, primarily through increased reactive oxygen species (ROS) and lactic acid production [21]. These findings suggested a potential association between ATPIF1 deficiency and the development of bacterial infection-associated colitis. While ATPIF1 is recognized as a regulator of cellular energy metabolism and inflammation, its specific roles in host defense and mucosal immunity during enteric bacterial infection remain unclear. To address this knowledge gap, the present study employed a *C. rodentium*-induced murine model to investigate the impact of ATPIF1 deficiency on colonic inflammation, inflammasome activation, and gut microbiota composition. Utilizing histopathological evaluation, quantification of inflammatory markers, and 16S rRNA gene sequencing, this research aims to elucidate the molecular and microbial mechanisms by which ATPIF1 contributes to infection-associated colitis pathogenesis, thereby highlighting its potential as a therapeutic target in bacterial infection-associated colitis.

## Materials and Methods

### Experimental Animals and Housing

The generation of ATPIF1 deficiency (ATPIF1^−/−^, KO) mice has been previously documented [20]. Male SPF C57BL/6 wild-type and ATPIF1^−/−^ mice aged 6 to 8 weeks were maintained under specific pathogen-free (SPF) conditions with a 12-h light/dark cycle, relative humidity of 40%–60%, and a temperature of 22 ± 2°C. Mice were provided with unrestricted access to food and sterile water. Following a 7-day acclimation period, experimental procedures were conducted. All animal protocols were reviewed and approved by the Institutional Animal Care and Use Committee (IACUC) of the College of Biological and Chemical Engineering (Approval No. CCSU-SYDWFLLL-002) and were performed in accordance with established institutional guidelines.

### Bacterial Culture and Induction of Colitis

The *Citrobacter rodentium* strain (ATCC 51459) was kindly supplied by Professor Zhiping Liu of Gannan Medical University. Frozen stocks of *C. rodentium* were inoculated into 5 mL of LB broth and incubated overnight at 37°C with agitation at 180 rpm. Subsequently, bacterial cultures were streaked onto MacConkey selective agar plates and incubated overnight. A single colony was then picked and transferred to fresh LB broth for activation [22]. The mice were randomly assigned to four groups (*n* = 5 per group): WT (wild type) group, WT+CR (*C. rodentium*-induced WT colitis) group, KO (ATPIF1^−/−^) group and KO+CR (*C. rodentium*-induced ATPIF1^−/−^ colitis) group. The experimental duration was 7 days. Mice in the WT+CR and KO+CR groups received a daily oral gavage of 200 μL of *C. rodentium* suspension (6 × 10^8^ CFU/mouse) [23]. Control groups (WT and KO) were administered an equivalent volume of sterile phosphate-buffered saline (PBS) via oral gavage. At the conclusion of the study (day 7), all mice were euthanized for further analysis.

### Body Weight Monitoring and Disease Activity Index (DAI)

Body weight was measured daily, and changes in weight were expressed as a percentage relative to the baseline body weight recorded on day 0. Disease Activity Index (DAI) scores were evaluated according to the criteria established by Murthy *et al*. [24], incorporating parameters such as body weight loss, stool consistency, and the presence of fecal occult blood. Rectal bleeding was assessed using a fecal occult blood test kit (Baso Biotechnology Co., Ltd., China). The overall DAI score was calculated as the mean of the three individual component scores.

### Fecal Sample Collection and Colon Length Measurement

On the seventh day, fresh fecal samples were collected and promptly stored at -80°C until further analysis. After euthanasia by cervical dislocation, the colon, including the anal segment, was carefully dissected, placed on a white background, and photographed. The length of the colon was then measured using a ruler.

### 16S rRNA Sequencing of Fecal Microbiota

Genomic DNA was isolated from fecal specimens using the Soil DNA Isolation Kit (Omega BioTek, USA) according to the manufacturer’s instructions. The quality and integrity of the extracted DNA were evaluated by electrophoresis on a 0.8% agarose gel. DNA concentration and purity were measured spectrophotometrically at 260 nm and 280 nm wavelengths using a NanoDrop 2000 spectrophotometer (Thermo Scientific, USA). DNA samples that met quality criteria were stored at -20°C for subsequent analyses.

To characterize the microbial community, the V3–V4 hypervariable region of the bacterial 16S rRNA gene was amplified using the primer pair 338F (5'-ACTCCTACGGGAGGCAGC-3') and 806R (5'-GGACTACHVGGGTWTCTAAT-3'). PCR products were purified using the QIAquick Gel Extraction Kit (QIAGEN, Germany), quantified, and normalized to uniform concentrations. Paired-end sequencing (2 × 300 bp) was performed on the Illumina MiSeq platform by MajorBio Bio-Pharm Technology Co., Ltd. (China). The raw sequencing data have been deposited in the NCBI Sequence Read Archive (SRA) under accession numbers SRR35154250 to SRR35154272.

### H&E and Alcian Blue Staining of Colon Tissue

Tissue samples from the distal colon, obtained proximal to the anal verge, were fixed in 4% paraformaldehyde. Subsequently, the specimens were dehydrated through a graded ethanol series, followed by clearing with xylene. The tissues were then embedded in paraffin and sectioned at a thickness of 5 μm. The resulting sections were stained with hematoxylin and eosin (H&E) as well as Alcian Blue.

### Histological Scoring and Quantification of Goblet Cells

Histological scoring of H&E-stained colon sections was conducted using the criteria proposed by Johansson [25], encompassing five parameters: inflammatory cell infiltration, mucosal thickening, goblet cell depletion, crypt loss, and epithelial damage. Each parameter was assigned a severity score ranging from 0 to 4, and the overall histological score was calculated by summing these individual scores. Additionally, Alcian Blue-stained sections were quantitatively analyzed using ImageJ software to measure the area exhibiting positive goblet cell staining.

### RNA Extraction and RT-qPCR

Total RNA was isolated from colon tissues using TRIzol reagent (Takara, China). Subsequently, cDNA was synthesized using PrimeScript RT Master Mix and stored at -80oC for further analysis (Takara). Quantitative real-time PCR was performed on a StepOne™ Real-Time PCR System (Life Technologies, USA) using SYBR Green PCR Master Mix (Takara) to assess gene expression. Expression levels of key inflammatory cytokines, including TNF-α, IL-1β, and IL-6, as well as the epithelial tight junction marker ZO-1, were quantified using gene-specific primers. GAPDH served as the internal control. Relative gene expression was calculated using the 2^−ΔΔCt^ method. Primer sequences used in this study are listed in the Table S1.

### Immunohistochemistry Analysis of Colon Tissue

Paraffin-embedded tissue sections were first deparaffinized and subjected to antigen retrieval via microwave heating. Endogenous peroxidase activity was inhibited using hydrogen peroxide (H_2_O_2_), followed by a 30-minute blocking step with ready-to-use goat serum (BosterBio, China). Subsequently, the sections were incubated overnight at 4°C with primary polyclonal antibodies against Cleaved Caspase-1 IgG, ASC IgG, and NLRP3 IgG, each diluted 1:200 (Wanleibio, China). After equilibration to room temperature, the sections were treated with appropriate secondary antibodies and visualized using a diaminobenzidine (DAB) detection kit (ShareBio, China). Nuclear counterstaining was performed with hematoxylin. Following dehydration and clearing through graded ethanol and xylene, the sections were mounted using neutral resin. Imaging was conducted with a fluorescence microscope (Leica, Germany) under standardized exposure conditions. Quantitative analysis of the immunohistochemically positive areas was performed using ImageJ software.

### Statistical Analysis

Statistical analyses were performed using GraphPad Prism version 5.0 (USA). Data are presented as the mean ± standard error of the mean (SEM). Differences between two groups were assessed using an unpaired Student’s *t*-test, while comparisons among multiple groups were conducted using one-way analysis of variance (ANOVA). A *p*-value less than 0.05 was considered statistically significant.

## Results

### ATPIF1 Deficiency Attenuates Disease Severity in *C. rodentium*-Induced Colitis

**Fig. 1:**
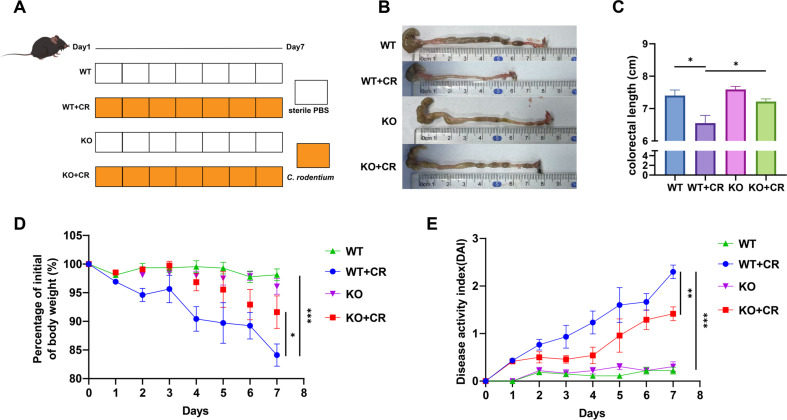
ATPIF1 deficiency attenuates colitis-associated symptoms in a *C. rodentium*-induced colitis model. (**A**) Schematic of the experimental timeline for the *C. rodentium*-induced colitis experiment. (**B**) Representative colon images harvested at the experimental endpoint. (**C**) Colorectal length (cm) measured at the experimental endpoint. (**D**) Daily body weight monitored throughout the experimental period. (**E**) Disease Activity Indices (DAI) scores assessed daily. WT: wild type; WT+CR: *C. rodentium*-induced WT colitis; KO: ATPIF1^−/−^; KO+CR: *C. rodentium*-induced ATPIF1^−/−^ colitis. Data are presented as mean ± SEM (*n* = 5). *p < 0.05, **p < 0.01, ***p < 0.001.

To assess colitis severity, we evaluated colorectal length, body weight loss, and disease activity index (DAI) scores across experimental groups. The experimental timeline for *C. rodentium*-induced colitis is shown in Fig. 1A. As illustrated in Fig. 1B and 1C, colon shortening in the KO+CR group was significantly less pronounced than in the WT+CR group (*p* < 0.05). Additionally, the KO+CR group exhibited significantly less weight loss compared to the WT+CR group (Fig. 1D, *p* < 0.05). Correspondingly, DAI scores were markedly lower in the KO+CR group than in WT+CR mice (Fig. 1E, *p* < 0.01). Collectively, these findings indicate that ATPIF1 deficiency attenuates the severity of *C. rodentium*-induced colitis.

### ATPIF1 Deficiency Reduces *C. rodentium* Burden in Feces and Colon Tissue

**Fig. 2:**
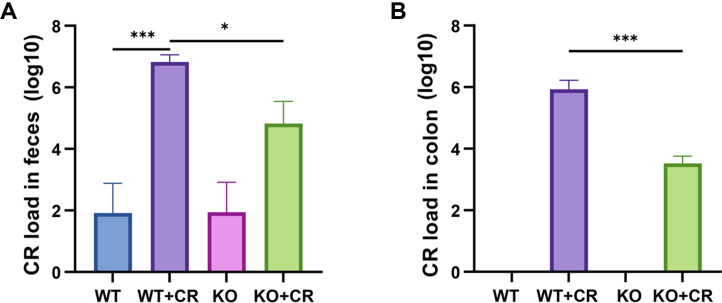
*C. rodentium* load in the feces and colon. (**A**) Bacterial load in feces (CFU/g) and (**B**) colon tissue (CFU/g) were quantified at the experimental endpoint to assess pathogen clearance capacity. WT: wild type; WT+CR: *C. rodentium*-induced WT colitis; KO: ATPIF1^−/−^; KO+CR: *C. rodentium*-induced ATPIF1^−/−^ colitis. Data are presented as mean ± SEM (*n* = 5). *p < 0.05, ***p < 0.001.

To assess the impact of ATPIF1 deficiency on bacterial load, we quantified *C. rodentium* colonization in fecal samples and colonic tissues using MacConkey agar selective medium at the study endpoint. As shown in Fig. 2A, fecal *C. rodentium* levels were significantly elevated in the WT+CR group compared to WT controls (*p* < 0.001), whereas the KO+CR group exhibited a significant reduction relative to WT+CR mice (*p* < 0.05). Similarly, bacterial load within colon tissues was markedly decreased in KO+CR mice compared to WT+CR counterparts (Fig. 2B, *p* < 0.001). These findings suggest that ATPIF1 deficiency enhances the host’s ability to clear opportunistic pathogens during *C. rodentium*-induced colitis.

### ATPIF1 Deficiency Alleviates Tissue Damage and preserves Mucosal Barrier Integrity in *C. rodentium*-Induced Colitis

**Fig. 3:**
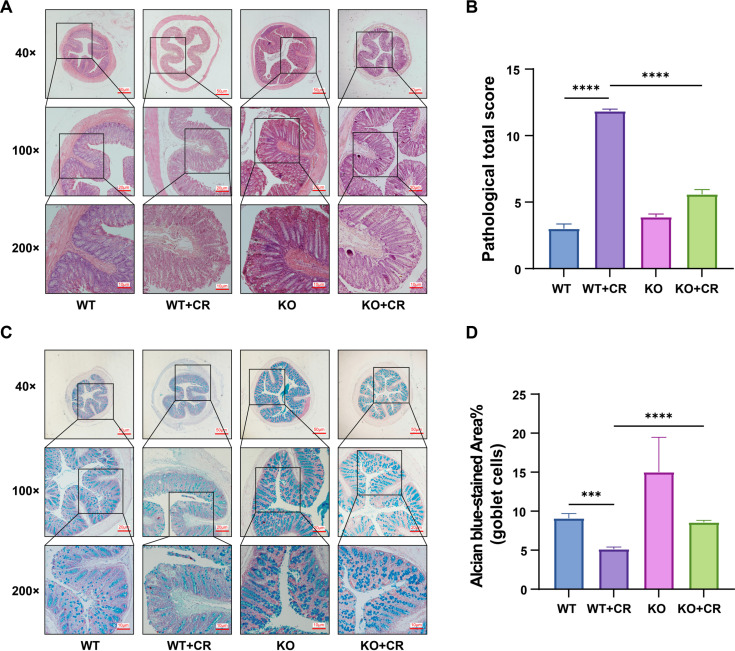
ATPIF1 deficiency suppressed colonic inflammation and maintained the integrity of the colonic mucosal barrier in *C. rodentium*-induced colitis mice. (**A**) Representative images of Hematoxylin and Eosin (H&E) staining of colonic tissue sections. (**B**) Pathological scores based on the H&E staining. (**C**) Representative images of colonic tissue sections stained with Alcian Blue. (**D**) Statistical analysis of Alcian Blue-stained goblet cells. WT: wild type; WT+CR: *C. rodentium*-induced WT colitis; KO: ATPIF1^−/−^; KO+CR: *C. rodentium*-induced ATPIF1^−/−^ colitis. Data are presented as mean ± SEM (*n* = 5). ***p < 0.001, ****p < 0.0001.

Histological analyses using hematoxylin and eosin (H&E) and Alcian Blue staining were performed to assess tissue damage and mucosal barrier integrity. The pathological score in WT+CR mice was significantly elevated (12.5 ± 1.12) compared to WT controls (*p* < 0.0001), characterized by disrupted colonic epithelium, extensive inflammatory cell infiltration, and pronounced submucosal edema. In contrast, KO+CR mice exhibited a significantly lower pathological score (5.6 ± 0.75) compared to WT+CR mice (*p* < 0.0001), with preservation of epithelial architecture, reduced edema, and attenuated inflammation (Fig. 3A and 3B). In parallel, Alcian Blue staining revealed a significant decrease in goblet cell percentage in WT+CR mice (1.76% ± 0.16) versus WT controls (2.95% ± 0.18, *p* < 0.001), whereas KO+CR mice showed a significant increase in goblet cell abundance (4.32% ± 0.37) compared to WT+CR (*p* < 0.0001, Fig. 3C and 3D). These data indicate that ATPIF1 deficiency not only mitigates *C. rodentium*-induced pathological damage but also maintains mucosal barrier integrity by preserving goblet cell populations, thereby contributing to the attenuation of inflammatory responses.

### ATPIF1 Deficiency Suppresses Inflammatory Cytokine Expression in *C. rodentium*-Induced Colitis

**Fig. 4:**
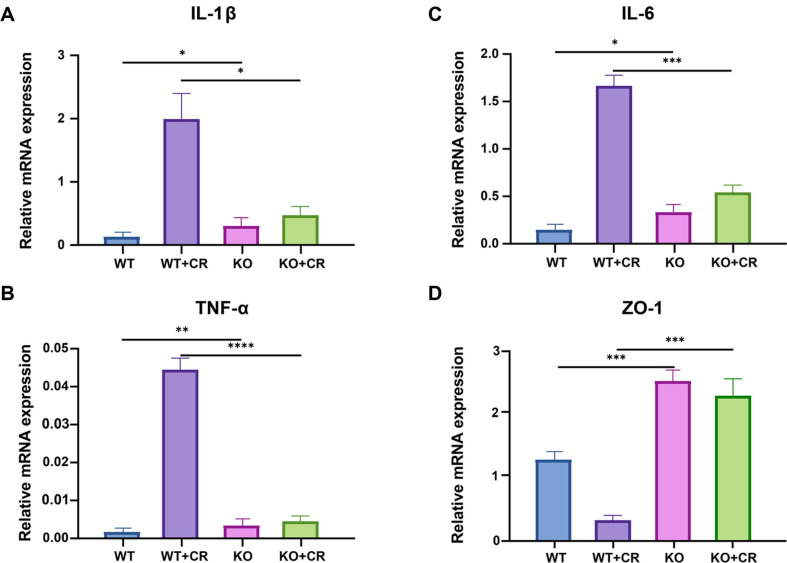
The relative mRNA expression levels of inflammatory cytokines and tight junction protein in colonic tissues. Relative mRNA expression levels of pro-inflammatory cytokines (**A**) IL-6, (**B**) IL-1β, (**C**) TNF-α, and (**D**) the tight junction protein ZO-1 in colonic tissues. WT: wild type; WT+CR: *C. rodentium*-induced WT colitis; KO: ATPIF1^−/−^; KO+CR: *C. rodentium*-induced ATPIF1^−/−^ colitis. Data are presented as mean ± SEM (*n* = 5). *p < 0.05, **p < 0.01, ***p < 0.001, ****p < 0.0001.

To further investigate the impact of ATPIF1 deficiency on intestinal inflammatory responses, we measured the relative mRNA abundance of key inflammatory cytokines in the colonic tissue of *C. rodentium*-induced colitis mice. As shown in Fig. 4A-4C, the expression levels of IL-6 (0.64 ± 0.08), IL-1β (0.46 ± 0.20), and TNF-α (0.0038 ± 0.0013) in the KO+CR group were significantly lower than those in the WT+CR group (*p* < 0.001, *p* < 0.05 and *p* < 0.0001, respectively). Additionally, as illustrated in Fig. 4D, the relative abundance of the tight junction protein ZO-1 in the KO+CR group (2.35 ± 0.37) was significantly higher than that in the WT+CR group (0.41 ± 0.09, *p* < 0.001). These findings suggest that ATPIF1 deficiency ameliorates colonic injury and preserves mucosal integrity, likely by maintaining goblet cell numbers and attenuating inflammatory cytokine production.

### ATPIF1 Deficiency Suppresses the Expression of NLRP3 Signaling-Associated Proteins in *C. rodentium*-Induced Colitis

**Fig. 5:**
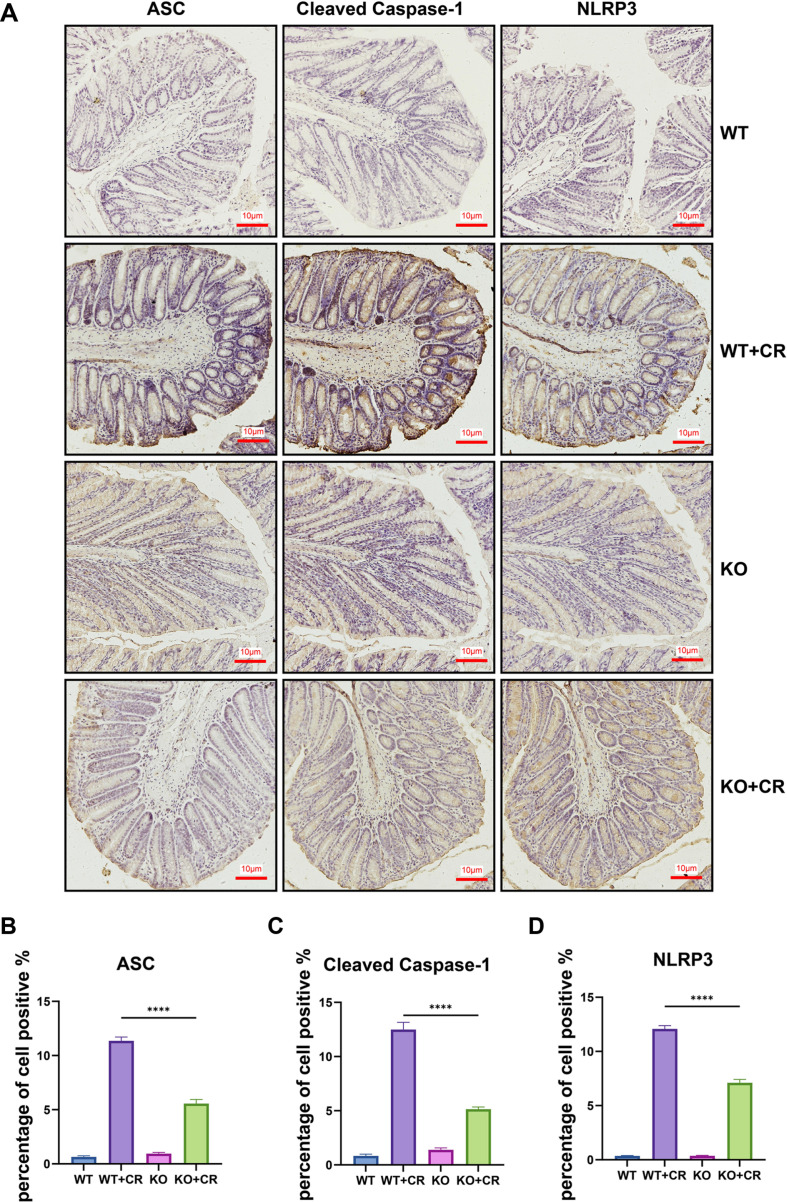
ATPIF1 deficiency suppressed the NLRP3 signaling pathway in *C. rodentium*-induced colitis mice. (**A**) Representative images of NLRP3 signaling pathway-related proteins ASC, Cleaved Caspase-1, and NLRP3 detected by immunohistochemistry. (**B-D**) Quantification of the percentage of cells positive for ASC, Cleaved Caspase-1, and NLRP3 in (**A**). WT: wild type; WT+CR: *C. rodentium*-induced WT colitis; KO: ATPIF1^−/−^; KO+CR: *C. rodentium*-induced ATPIF1^−/−^ colitis. Data are presented as mean ± SEM (*n* = 5). ****p < 0.0001.

Based on the observed downregulation of IL-6, IL-1β, and TNF-α in the KO+CR group, we next investigated whether ATPIF1 deficiency modulates the NLRP3 inflammasome signaling pathway. The NLRP3 inflammasome recognizes pathogen-associated molecular patterns (PAMPs) and damage-associated molecular patterns (DAMPs), assembling with ASC and pro-caspase-1 to form a multiprotein complex that activates caspase-1 [26], leading to the maturation and secretion of IL-1β and IL-18 [13]. TNF-α can enhance NLRP3 activation and the inflammatory response, with these inflammatory factors constituting a pro-inflammatory positive feedback loop [12]. To investigate whether ATPIF1 deficiency alleviates intestinal inflammatory responses in the colon of *C. rodentium*-induced colitis mice via the NLRP3 signaling pathway, we measured the expression of NLRP3 signaling-related proteins ASC, Cleaved Caspase-1, and NLRP3. The results showed that the inflammasome signaling pathway was abnormally active in the WT+CR group, with markedly increased expression of ASC, Cleaved Caspase-1, and NLRP3 (Fig. 5A-5D). In contrast, the expression levels of ASC, Cleaved Caspase-1, and NLRP3 were significantly decreased in the KO+CR group (Fig. 5A-5D, *p* < 0.0001). These findings suggest that ATPIF1 deficiency is associated with reduced NLRP3 inflammasome activation in *C. rodentium*-induced colitis, although whether this reflects a direct suppressive effect of ATPIF1 on inflammasome signaling or a consequence of attenuated inflammatory burden remains to be determined.

### ATPIF1 Deficiency Alleviates Gut Microbiota Dysbiosis in *C. rodentium*-Induced Colitis in Mice

**Fig. 6:**
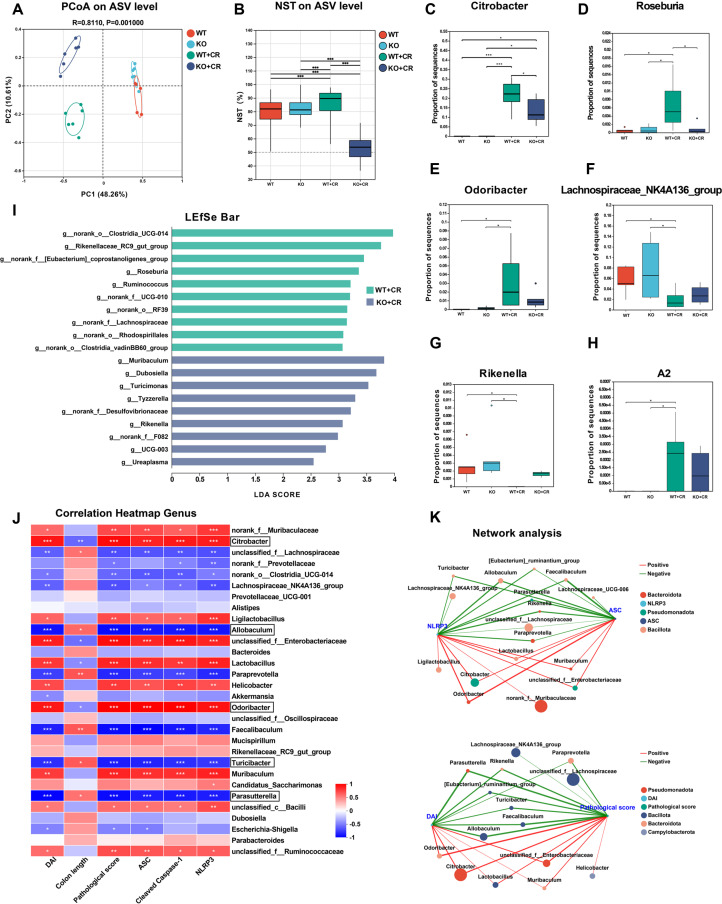
Modulation of gut microbiota by ATPIF1 deficiency in *C. rodentium*-induced colitis mice. (**A**) Principal Coordinates Analysis (PCoA). (**B**) Normalized Stochasticity Ratio (NST) analysis. (**C–H**) Relative abundances of some characteristic genera. (**I**) Linear Discriminant Analysis Effect Size (LEfSe) analysis. (**J**) Correlation analysis between the top 30 abundant genera and micro-environmental factors. (**K**) Network analysis of genus-level gut microbiota with inflammatory pathway component and clinical indicators. WT: wild type; WT+CR: *C. rodentium*-induced WT colitis; KO: ATPIF1^−/−^; KO+CR: *C. rodentium*-induced ATPIF1^−/−^ colitis. Data are presented as mean ± SEM (*n* = 5). *p < 0.05, **p < 0.01, ***p < 0.001.

Gut microbiota dysbiosis is implicated in the pathogenesis of infection-associated colitis. To assess the influence of ATPIF1 deficiency on the gut microbial composition in mice with *C. rodentium*-induced colitis, we performed high-throughput sequencing of the 16S rRNA gene from mouse fecal samples. No significant difference was observed in alpha diversity indices among the WT, WT+CR, KO, and KO+CR groups. However, beta-diversity analysis revealed marked alterations in the gut microbiota composition. Principal coordinates analysis (PCoA) based on Bray-Curtis distances at the ASV level demonstrated that the microbial communities of the KO and WT groups partially overlapped, although distinct differences were observed. In contrast, after *C. rodentium* infection, the microbial compositions of WT+CR and KO+CR groups were distinctly separated from each other, indicating substantial structural divergence in the gut microbiota between these groups (Fig. 6A). The normalized stochasticity ratio (NST) values indicated that gut microbial assembly in both WT and KO groups under basal conditions was predominantly governed by stochastic processes (Fig. 6B). The influence of stochasticity was increased in WT+CR group (86.03 ± 9.62%), suggesting disrupted gut environmental selection (*p* < 0.001). Conversely, the NST value in the KO+CR group significantly decreased to 53.19 ± 8.65%, indicating a shift in microbial community assembly toward more deterministic processes. This shift may be attributed to the alteration of the host intestinal environment caused by ATPIF1 deficiency, whereby enhanced host-mediated selection pressure potentially reflects a more structured and regulated microbial composition.

At the genus level, the microbial community composition revealed distinct differences among groups (Fig. S1A). After *C. rodentium*-induced colitis, the abundances of *Citrobacter* and *Roseburia* were significantly reduced in the KO+CR group (Fig. 6C and 6D, *p* < 0.05), while *Odoribacter* was significantly enriched in the WT+CR group (Fig. 6E, *p* < 0.05). In contrast, *Lachnospiraceae_NK4A136_group* and *Rikenella* were markedly decreased in the WT+CR group, with no significant reduction observed in the KO+CR group (Fig. 6F and G, *p* < 0.05). Notably, a genus designated A2, which was rarely reported, was significantly enriched in the WT+CR group (Fig. 6H).

Characteristic genera of each group were further identified using linear discriminant analysis coupled with effect size (LEfSe). As shown in Fig. 6I, unclassified *Clostridia_UCG-014*, *Rikenellaceae_RC9*_gut_group, *Roseburia*, and *Ruminococcus* were identified as signature taxa of the WT+CR group, while *Muribaculum*, *Dubosiella*, *Turicimonas*, *Tyzzerella*, and *Rikenella* were the characteristic genera enriched in the KO+CR group.

The correlations between the top 30 most abundant genera and various environmental factors, including DAI score, colon length, pathological score, and key components of the NLRP3 signaling pathway (ASC, cleaved Caspase-1, and NLRP3), were analyzed to explore the relationship between gut microbiota and host-associated inflammatory parameters (Fig. 6J). Colon length showed positive correlations with *Parasutterella*, *Turicibacter*, and *Lachnospiraceae_NK4A136*_group, but was negatively correlated with *Citrobacter*, *Rikenellaceae_RC9*_gut_group, and *Odoribacter*. In contrast, indicators of colitis severity, including DAI score, pathological score, and NLRP3 pathway-related proteins, were positively associated with *Citrobacter*, *Odoribacter*, and *unclassified_f__Enterobacteriaceae*, and negatively associated with *Parasutterella*, *Dubosiella*, and *Turicibacter*. The network analysis between the top 50 most abundant genera and environmental factors also showed that the severity of colitis was positively related to *Citrobacter* and *Odoribacter*, while negatively related to *Allobaculum*, *Turicibacter*, *Paraprevotella* and *Rikenella* (Fig. 6K). It should be noted that the functional contributions of these bacterial taxa to the observed protective phenotype have not been directly examined in the present study, and future investigations using gnotobiotic models or fecal microbiota transplantation will be required to determine whether these genera directly contribute to disease protection.

Predicted functional analysis of microbial metabolic pathways revealed that, at KEGG level 3, Fatty acid biosynthesis, the AMPK signaling pathway and the NOD-like receptor signaling pathway were significantly downregulated in the KO+CR group compared with those in the WT+CR group (*p* < 0.05). Interestingly, in the COG functional classification, the ATPase activity pathway was also markedly suppressed in the KO+CR group (*p* < 0.05). The reduced ATPase activity observed in KO mice may reflect a mitochondrial adaptation that stabilizes the membrane potential without requiring excessive ATP hydrolysis (Fig. S1B and S1C).

## Discussion

The global incidence of ulcerative colitis (UC) has risen substantially, underscoring the critical need for effective preventive and therapeutic interventions. UC is a complex disorder influenced by genetic predisposition, immune system dysregulation, and environmental factors [27]. Emerging evidence demonstrates significant differences in gut microbiota composition between UC patients and healthy controls, positioning the microbiota as a pivotal mediator in host-pathogen interactions during disease pathogenesis [28]. Although our previous research demonstrated that ATPIF1 deficiency effectively ameliorates DSS-induced colitis [20], its role in infection-driven intestinal inflammation and gut microbiota regulation remains inadequately characterized. In the present study, utilizing a *C. rodentium* infection-induced colitis model, we observed that ATPIF1 deficiency significantly attenuates *C. rodentium*-induced colitis. This protective effect correlates with suppression of the NLRP3 inflammasome pathway and preservation of gut microbial homeostasis, thereby identifying ATPIF1 as a promising therapeutic target in infection-associated colitis.

*C. rodentium* is a natural murine attaching and effacing (A/E) pathogen capable of inducing transmissible colonic hyperplasia [29]. Pathogen invasion is closely associated with infectious diarrhea and colitis, with *C. rodentium*-infected mice exhibiting pronounced colitis symptoms, including weight loss, colon shortening, and fecal occult blood [28]. Importantly, *C. rodentium* infection does not require antibiotic pretreatment, establishing it as a primary model for studying A/E pathogen-induced intestinal inflammation [30]. In C57BL/6 mice, *C. rodentium* infection progresses through four stages: *Establishment*, with *C. rodentium* colonization in cecal patches; *Expansion*, characterized by distal colon colonization, crypt hyperplasia, and epithelial barrier disruption; *Steady-state*, with stable *C. rodentium* shedding (~10^9^ CFU/g feces) accompanied by neutrophil and Th17/Th22 cell-mediated IL-17A and IL-22 secretion, the absence of which is lethal; and *Clearance*, wherein CD4^+^ T and B lymphocytes shift from IL-17A/IL-22 to IFN-γ-dominated responses, and IFN-γ deficiency delays pathogen clearance. *C. rodentium* is ultimately eliminated through IgG-mediated opsonization, neutrophil phagocytosis, and competition with commensal bacteria. As an initial host defense mechanism, activated intestinal epithelial cells secrete antimicrobial peptides, serum amyloid A (SAA), and reactive oxygen species (ROS) to restrict bacterial adherence [31]. Innate immune recognition is further mediated by Toll-like receptors (TLRs), intracellular NOD-like receptors (NLRs), and signals from damaged epithelial cells, collectively orchestrating infection sensing and response [32]. Adaptive immunity, particularly involving CD4^+^ T and B lymphocytes, is essential for pathogen clearance[33]. *C. rodentium* infection elicits robust innate immune activation, with the NLRP3 inflammasome serving as a central signaling nexus, as evidenced by the elevated activation of NLRP3 inflammasome signaling observed in the WT+CR group in the present study.

Lipopolysaccharide (LPS) derived from *C. rodentium* is detected by epithelial and immune cells, including macrophages and dendritic cells, activating the TLR4/MyD88/NF-κB signaling cascade to induce expression of NLRP3, pro-IL-1β, and pro-IL-18, thereby priming inflammasome assembly [34]. Additionally, *C. rodentium* utilizes a Type III Secretion System (T3SS) to translocate effector proteins that disrupt mitochondrial function and compromise tight junction integrity [35]. Excessive ROS production, originating from mitochondria and NADPH oxidase, further promotes NLRP3 activation. The inflammasome complex, comprising NLRP3, ASC, and pro-Caspase-1, facilitates the secretion of IL-1β and IL-18, recruitment of neutrophils, and propagation of intestinal inflammation. NLRs also mediate pyroptosis, an inflammatory form of programmed cell death in myeloid cells [19]. Our data demonstrate that ATPIF1 deficiency is associated with NLRP3 signaling in mice, accompanied by preservation of mucosal architecture and attenuation of inflammatory infiltration as observed in the present study (Fig. 5A-5D). However, whether this reduction reflects a direct suppressive effect or a consequence of reduced pathogen burden remains to be determined.

Previous studies have indicated that NLRP3 activity is negatively regulated by autophagy but positively modulated by reactive oxygen species (ROS) derived from damaged organelles [21]. Mitochondrial dysfunction leads to ROS accumulation, thereby linking organelle impairment to inflammasome-mediated inflammation. Mitochondrial ATP synthesis predominantly depends on F1Fo-ATP synthase, whose activity is inhibited by ATPIF1 overexpression, resulting in increased oxidative stress and amplified inflammatory responses [20]. Consistent with this, ATPIF1 deficiency reduced pro-inflammatory cytokine levels in our study (Fig. 4A-4C). Moreover, prior investigations employing peritonitis models revealed that ATPIF1 loss enhances glycolytic flux and augments neutrophil antimicrobial activity *via* ROS and lactate production [21], which may partially account for the reduced *C. rodentium* burden observed in KO+CR mice and its downstream inflammatory consequences. It should be noted that the present study does not fully distinguish between the direct and indirect effects of ATPIF1 deficiency on mucosal inflammation and barrier function. However, direct modulation of mucosal immunity by ATPIF1 deficiency is also supported by findings from DSS-induced colitis models [20], as well as by the preservation of goblet cell numbers and mucosal barrier integrity observed in the present study. Together, both epithelial protection and enhanced neutrophil antimicrobial function may collectively contribute to disease amelioration. Future studies, including germ-free or antibiotic-pretreated models, will be required to further dissect the relative contributions of direct immune regulation and indirect pathogen clearance.

Colitis progression is frequently accompanied by alterations in gut microbiota composition. While α-diversity remained unchanged, β-diversity differed significantly between the WT+CR and KO+CR groups, suggesting that ATPIF1 deficiency reshapes bacterial community composition without affecting richness or evenness. Normalized Stochasticity Ratio (NST) analysis revealed that microbial assembly in KO+CR mice shifted toward a more deterministic pattern, indicating preferential recovery of key functional taxa that may promote SCFA metabolism and enhance host resistance to stress and pathogens. In contrast, microbial assembly in the WT+CR group was primarily governed by stochastic processes. Correlation analyses between bacterial genera and inflammasome components further support bidirectional interactions: microbial metabolites may modulate NLRP3 activation, while inflammation-induced environmental changes can promote the expansion of pathogenic taxa. Under *C. rodentium*-induced stress, ATPIF1-deficient mice maintained beneficial taxa such as *Dubosiella*, *Rikenella*, and *Parasutterella* compared to WT mice. *Dubosiella* produces short-chain fatty acids (SCFAs) to balance Treg/Th17 responses and improve mucosal barrier integrity, and to enhance immune tolerance through the IDO1-Kynurenine pathway. *Rikenella* is considered a potential probiotic, while *Parasutterella* is a core component of murine and human gut microbiota, involved in succinate productiona space before the citation [3, 36, 37]. Preservation of these taxa likely contributes to microbial stability in KO+CR mice. However, the functional contributions of these taxa to the observed protective phenotype have not been directly examined in the present study. Future investigations using gnotobiotic models or fecal microbiota transplantation will be required to determine whether these genera directly contribute to disease protection. Downregulation of fatty acid metabolism at the KEGG Level 3 pathway indicates reduced inflammation and stabilized microbial composition, whereas suppression of the AMPK pathway reflects diminished cellular stress and inflammatory state, suggesting reduced demand for intensive metabolic regulation [38]. Similarly, NOD-like receptor pathway activity aligns with inflammasome status [39]. Additionally, ATP synthase activity was suppressed in KO mice, suggesting a potential mitochondrial energy-conserving adaptation. Collectively, these findings imply that ATPIF1 deficiency is associated with altered host metabolism and microbial community remodeling, though whether gut microbiota changes directly mediate the protective effects of ATPIF1 deficiency remains to be established.

Despite observing reduced expression of NLRP3 inflammasome-related proteins, upstream mechanisms—including mitochondrial ROS generation, ASC speck formation, and TLR4 activity—warrant further elucidation. Functional validation of specific microbial taxa, such as *Roseburia*, will be instrumental in clarifying their contributions to the protective phenotype observed in ATPIF1-deficient mice.

In summary, this study demonstrates that ATPIF1 deficiency exhibited a protective phenotype in *C. rodentium*-induced colitis, which was accompanied by reduced NLRP3 inflammasome activation and stabilization of gut microbial composition. These findings are consistent with our previous observations in the DSS model and suggest that ATPIF1 deficiency is associated with attenuated disease severity across distinct colitis contexts [20]. We acknowledge, however, that the direct causal relationships among ATPIF1 deficiency, NLRP3 inflammasome suppression, microbiota remodeling, and disease alleviation have not been formally established in the present study and represent an important direction for future investigation. Nonetheless, these results identify ATPIF1 as a potentially relevant node at the intersection of mitochondrial metabolism, innate immune signaling, and microbial homeostasis, with implications for the development of metabolic therapeutic strategies in infection-associated colitis.

## Supplemental Materials

Supplementary data for this paper are available on-line only at http://jmb.or.kr.
