# Supplementary material for: ATPIF1 Deficiency Significantly Alleviates Citrobacter rodentium-Induced Colitis in Mice
Source: J Microbiol Biotechnol. 2026 May 29;36:e2604015. doi: 10.4014/jmb.2604.04015 (PMC13249600; doi:10.4014/jmb.2604.04015)
Supplement: Supplementary file 1 [file jmb-36-e2604015-supple.pdf]

J. Microbiol. Biotechnol. 2026. 36: e2604015
https://doi.org/10.4014/jmb.2604.04015

Supplementary Figure and Table

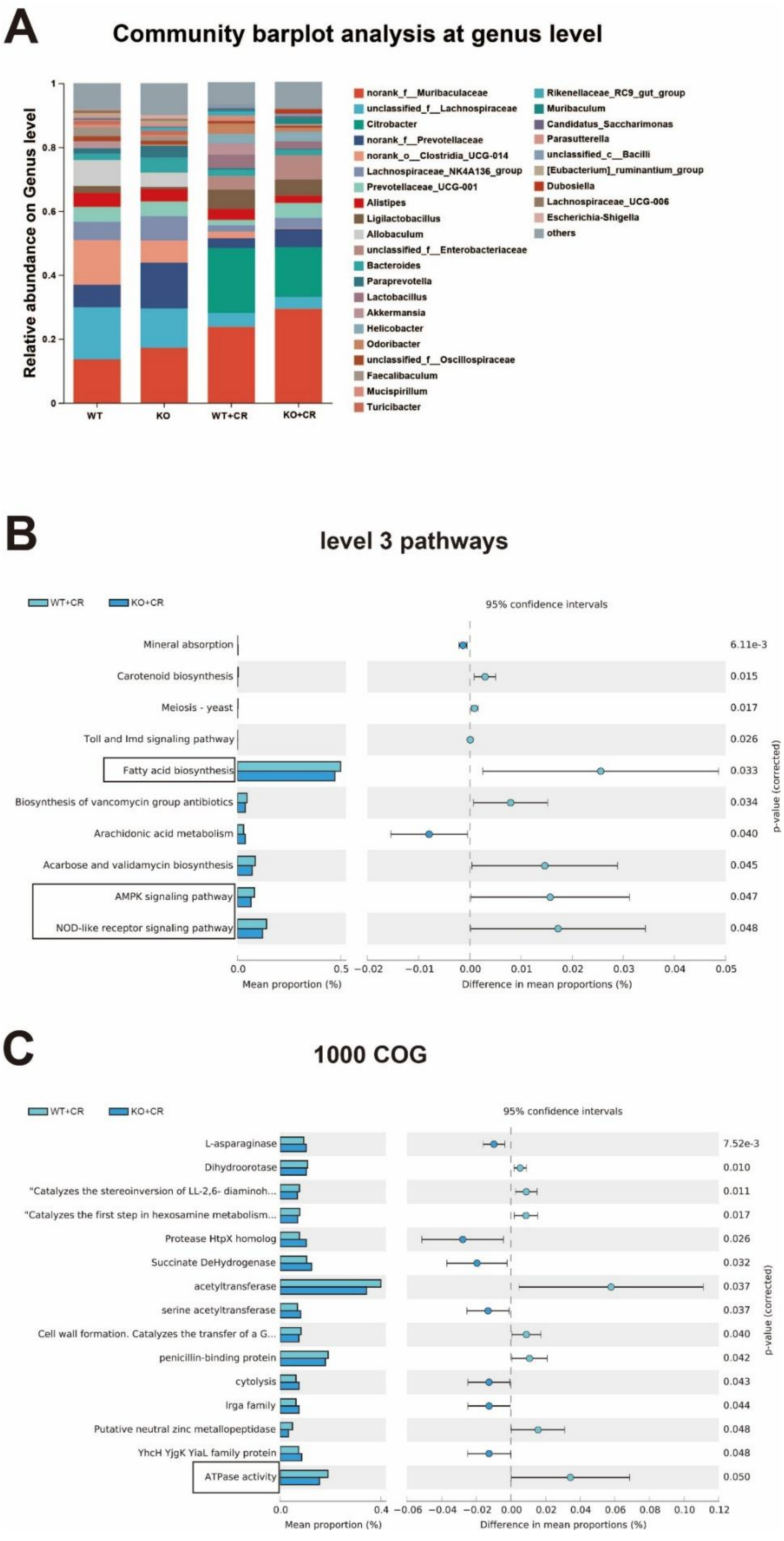

Fig. S 1. (**A**) Bacterial composition at genus level; Prediction of 16S rRNA gene function based on KEGG at level 3 (**B**) and the most abundant 1000 COG functions (**C**). WT: wild type; WT+CR: *C. rodentium*-induced WT colitis; KO: ATPIF1$^{-/-}$; KO+CR: *C. rodentium*-induced ATPIF1$^{-/-}$ colitis.

Table S1. Primer sequences in RT-qPCR

Items	Forward primer (5'-3')	Reverse primer (5'-3')
TNF-α	CCCTCACACTCAGATCATCTTCT	GCTACGACGTGGGCTACAG
IL-1β	GCAACTGTTCCTGAACTCAACT	ATCTTTTGGGGTCCGTCAACT
IL-6	TAGTCCTTCCTACCCCAATTTCC	TTGGTCCTTAGCCACTCCTTC
ZO-1	GCCGCTAAGAGCACAGCAA	GCCCTCCTTTTAACACATCAGA
GAPDH	TCTGGAAAGCTGTGGCGTGAT	TCTGGAAAGCTGTGGCGTGAT
